# Grape seed extract alleviates radiation-induced damages in human blood lymphocytes

**Published:** 2020

**Authors:** Reza Ghasemnezhad Targhi, Valiallah Saba

**Affiliations:** 1 *Department of Radiology, Imam Reza Hospital, Mashhad University of Medical Sciences, Mashhad, Iran*; 2 *Department of Radiology, Faculty of Para medicine, AJA University of Medical Sciences, Tehran, Iran*

**Keywords:** Grape seed extract, Ionizing radiation, Micronucleus, Lymphocytes and radioprotective agents

## Abstract

**Objective:**

Ionizing radiation induces deleterious effects in the biological systems by producing free radicals. Grape Seed Extract (GSE) as a free radical scavenger could protect the body against the damages*.*

**Materials and Methods:**

In this study, 12 healthy male volunteers were divided into Groups 1, 2, 3 and 4 and received 100, 300, 600 and 1000 mg GSE, respectively. Peripheral blood samples were collected from each volunteer 15 min before, and 1, 2, and 5 hr after GSE oral administration. Blood samples were then irradiated with 150 cGy of 100 kvp X-ray (Irradiated control group, was treated with only 1.5 Gy of X-rays). Cytogenic damages were detected by micronucleus assay.

**Results:**

Results showed that irradiation significantly increased the incidence of micronuclei (p<0. 001). In group 1, the mean reduction of micronucleus rate was 26.53%, 34.92%, and 31.38%, 1, 2, and 5 hr after GSE ingestion (p<0.001), respectively; this variable in group 2 was 17.38, 38.33, and 31.38 (p<0. 001), in group 3, was 35.65%, 46%, and 37.15% (p<0.001), respectively and in group 4, was 41.35%, 51.73%, and 50.55% (p<0.0001), respectively. The samples collected 1, 2, and 5 hr after ingestion of GSE exhibited a significant decrease in the incidence of micronuclei compared with the radiation control group. The maximum protection and reduction in frequency of micronuclei (51.73%) was observed 2 hr after ingestion of 1000 mg GSE.

**Conclusion:**

Consumption of GSE before undergoing radiation protects human lymphocytes against X-rays by reducing radiation-induced genotoxicity.

## Introduction

Ionizing radiation causes deleterious effects in biological systems, mediated by oxidative stress induced by production offree radicals such as reactive oxygen species (ROS), hydroxyl radicals (OH) and superoxide (O) (Wang et al., 2018 ▶). The reaction between free radicals and cellular components like DNA, proteins, and membranes leads to DNA strand break, lipid peroxidation, and protein modification that cause mutation and cancer (Pisoschi and Pop, 2015 ▶; Vasilyeval and Bespalov, 2015 ▶). So, Radioprotector agents have been developed to protect humans against harmful effect of ionizing radiation. 

As mentioned, free radicals play a key role in radiation-induced cellular and genotoxic damages; so, any molecule with free-radicals scavenging properties, acts as a radioprotector (Smith et al., 2017 ▶). Amifostine is the only radioprotector that is approved by FDA and used in clinics. It has some side effects such as hypertension, vomiting, and nausea (Cheki et al., 2016 ▶). So, we need a potent radioprotector with lower toxicity and fewer side effects.

In the recent years, natural compounds have been noticed by the researchers as new and efficient radioprotectors. Plant products with antioxidant properties and free-radicals scavenging abilities, have superiority over synthetic compounds. In various animal studies, administration of different herbal extracts (Black mulberry, *Origanum vulgare*, Abana, Triphala, *Mangifera indica*, Panax ginseng, *Mentha piperita*, Green tea and Grape seed) before irradiation, resulted in low mortality rate and reduced the symptoms of radiation damages compared to the radiation control group (Targhi et al., 2017 ▶; Targhi et al., 2016 ▶; El-Desouky et al., 2017 ▶).

Positive effects of grape seed extract (GSE) on health are supposed to originate mainly from its antioxidant compounds. Grape seed are rich in flavonoids, phenolics, anthocyanin and proanthocyanidin (De Nisco et al., 2013 ▶). Grape seed pro-anthocyanidins showed anti-inflammatory, cardio-protective, immune modulating and anti-carcinogenic activities (Rice-Evans et al., 1996 ▶). The radioprotective effect of grape vine fruit extract was shown against radiation-induced oxidative stress and apoptosis in human lymphocytes (Singha and Das, 2015 ▶). Some reports confirmed that anthocyanins are good antioxidants, and they can effectively remove free radicals (Huang et al., 2014 ▶).

Radioprotective features of plant extracts are mainly studied by evaluating their ability to alleviate radiation-induced chromosomal aberrations and micronuclei formation (Hosseinimehr, 2007 ▶). Micronuclei may originate from chromosomal fragments or whole chromosome delay in the anaphase stage. In this study, genotoxic damages were evaluated by micronuclei assay that is a powerful method, in human blood lymphocytes. This assay has been widely used to investigate the effects of potential radioprotector agents. 

The present study evaluated protection effect of GSE against radiation-induced micronucleus in human blood lymphocytes.

## Materials and Methods

**Human blood samples**

This experimental study was registered with an IRCT20160204026361N2 number in Iranian Registry of Clinical Trial and approved by the Ethics Committee of Mashhad University of Medical Sciences, Mashhad, Iran. 

Informed consent forms were collected from 12 male healthy and non-smoking volunteers with the age of 20-30 years old. The volunteers had no history of pharmacotherapy and medical radiation exposure for at least two months before sampling and did not suffer from any serious acute or chronic diseases. The volunteers were divided into four groups. After overnight fasting, volunteers in group 1 ingested a single oral dose of 100 mg GSE (Ahvaz research herbal pharmaceutical center, Iran), volunteers in group 2 ingested 300 mg GSE, volunteers in group 3 ingested 600 mg GSE and volunteers in group 4 ingested 1000 mg GSE. The blood samples were collected in heparinized tubes 15 min before, and 1, 2, and 5 hours after ingestion of four doses of GSE. 

**Irradiation and cell culture for determination of micronuclei**

For each volunteer at each sampling time, 1 ml of heparinized blood was divided into two halves and poured into two 25 ml culture flasks. One culture flask was kept as a non-irradiated control sample and another one was irradiated with a 100 kvp X-ray (Omid hospital, Mashhad, Iran). The X-ray unit was calibrated for irradiating human whole blood samples. Irradiation was performed at a dose of 150 cGy at 37˚C. For irradiation, the flasks were placed on a 30×30×20 cm water phantom and covered with a 20×20 cm applicator which was attached to a superficial X-ray unit (Philips, serial number: 2/625, Amsterdam, Netherlands) , dose-rate was 1.51 Gy/min and SSD (source surface distance) was 30 cm. 

Subsequently, each sample (non-irradiated and irradiated) was added to 4.5 ml of RPMI 1640 culture medium (Sigma, USA) containing 20% fetal calf serum, 100 μl/ml phytohaemagglutinin (Sigma, USA), 100 μl/ml penicillin, 250 μg/ml streptomycin and 2 mM L-glutamine (Sigma, USA). All cultures were incubated at 37±1˚C in a humidified atmosphere of 5% CO_2_ and 95% O_2_. After 46 hr culture, cytochalasin B (Sigma, USA) at final concentration of 30 μl/ml, was added to the samples. After 72 hr incubation, cells were collected by centrifuging at 1000 rpm for 5 min.

The collected cells were suspended in 0.075 M cold potassium chloride (KCl) and centrifuged at 800 g (acceleration of gravity) for 6 min and instantly fixed in a fixative solution (methanol: acetic acid, 6:1) for 3 times. To prepare the microscopic slides, the fixed cells were dropped onto clean microscopic slides, air-dried and stained with Giemsa solution (Merck, Germany). For assessment of micronuclei the slides was carried out at X100 magnification. Criteria for scoring micronuclei were diameters between 1/16th and one-third of main nuclei, non-refractive, not linked to main nuclei and not overlapping the main nuclei (Fenech, 2000). At each blood sampling time, for each volunteer, from irradiated and control cultures, a total of 2000 binucleate cells (1000 cells each from duplicate cultures) were examined to record the micronucleus frequency.

**Statistical analysis**

All shown results are Mean±Standard deviation. SPSS 16.0 statistical analysis software was used for data analysis, and comparison between groups was made by One-way ANOVA analysis and a p-value<0.05 was considered to represent a statistically significant difference. 

## Results

**Frequency of micronuclei in human blood lymphocytes after administration of GSE**

**Figure 1:**
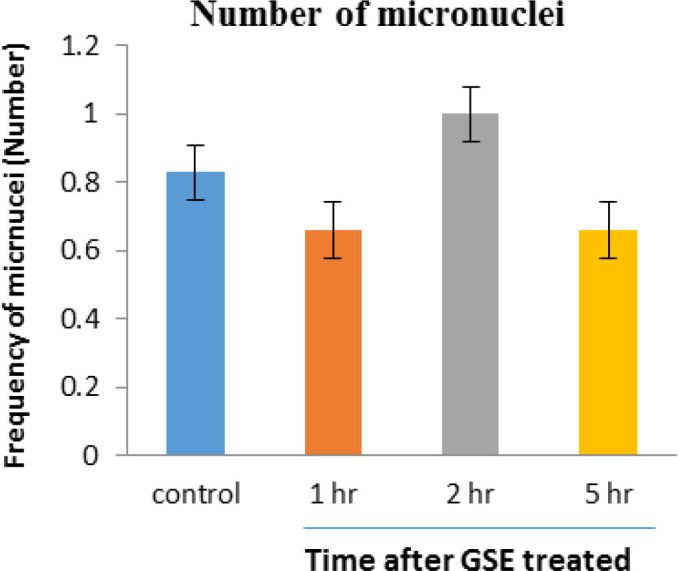
Mean number of micronucleated cells in human blood lymphocytes. Error bars indicate standard deviation(s). 1hr, 2hr and 5hr indicate frequency of micronuclei 1hr, 2hr and 5 hr after ingestion of 1000 mg GSE, respectively

No obvious side effect was reported by any volunteers. Data showed that the groups that received 1000 mg (maximum dose) GSE showed no significant difference in frequency of micronuclei in human blood lymphocytes compared with the control group (non-treated group). The mean frequency of micronuclei in the control group was 0.83 and 1, 2, and 5 hr after ingestion of GSE was 0.66, 1.00 and 0.66, respectively (Figure 1). 

**Figure 2:**
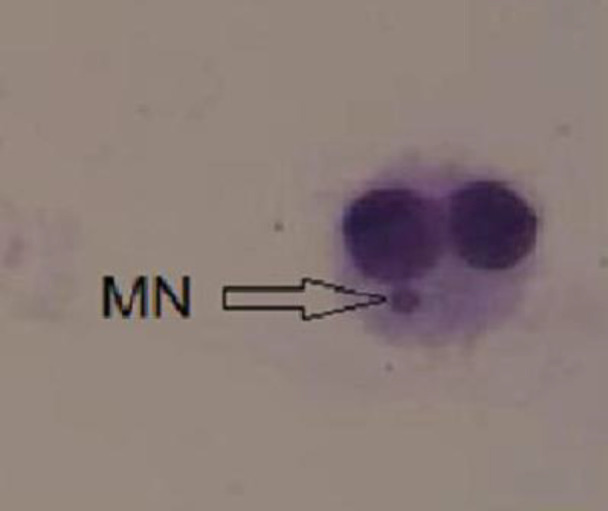
A typical binucleated lymphocyte with micronuclei

A typical human lymphocyte with micronuclei is shown in Figure 2.

**Frequency of micronuclei in human blood lymphocytes that were irradiated with 1.5 Gy X-radiation after administration of 100 mg GSE**

**Figure 3:**
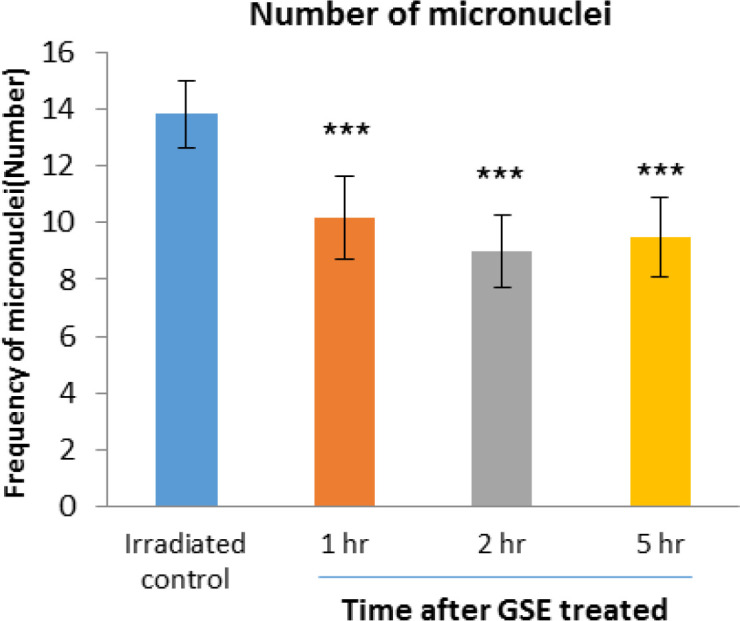
Mean number of micronucleated cells in human blood lymphocytes. Error bars indicate standard deviation(s) values. ***p<0.001 compared to irradiated control group. 1hr, 2hr and 5hr, indicate the groups, which were irradiation 1hr, 2hr and 5 hours after ingestion of 100 mg GSE

The frequency of micronuclei induced by 150 cGy of X-radiation in human lymphocyte in the control irradiated group was 13.83 and 1, 2 and 5 hr after 100 mg GSE ingestion were 10.16 (p<0.05), 9 (p<0.001), and 9.5 (p<0.001), respectively, compared to the irradiated control group. The frequency of micronuclei found in GSE-treated cell groups was significantly lower than the control irradiated group. For the blood samples collected 2 hr after the oral GSE administration and *in vitro *exposed to 150 cGy of X-ray radiation, more reduction (34.92) in the incidence of micronuclei was observed (p<0.01) (Figure 3). 

**Frequency of micronuclei in human blood lymphocytes that were irradiated with 1.5 Gy X-radiation after administration of 300 mg GSE**

**Figure 4:**
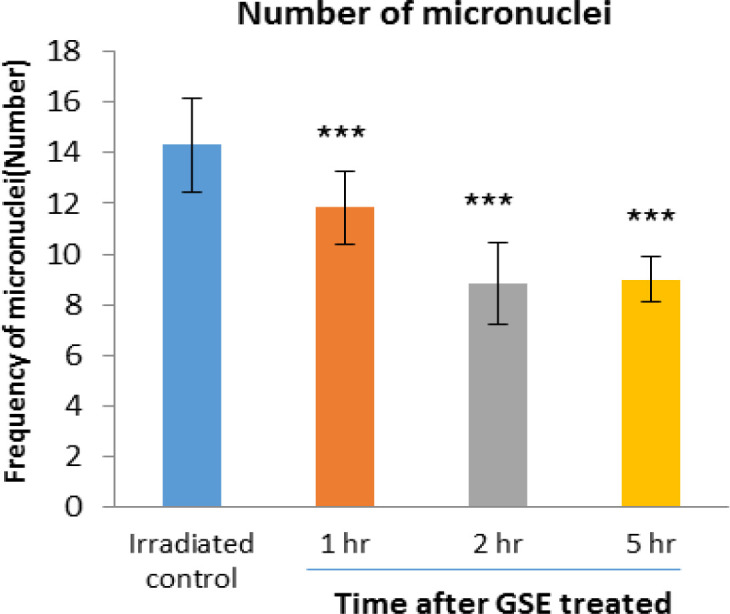
Mean number of micronucleated cells in human blood lymphocytes. Error bars indicate standard deviation(s) values. ***p<0.001 compared to irradiated control group. 1hr, 2hr and 5hr, indicate the groups, which were irradiation 1hr, 2hr and 5 hours after ingestion of 300 mg GSE

The frequency of micronuclei induced by 150 cGy of X-radiation in human lymphocyte in irradiated control group was 14.32 and 1, 2 and 5 hr after 300 mg GSE ingestion were 11.83 (p<0.001), 8.83 (p<0.001), and 9 (p<0.001), respectively, compared to the irradiated control group. The frequency of micronuclei found in GSE-treated cell groups was significantly lower than the control irradiated group. For the blood samples collected 2 hr after the oral GSE administration and *in vitro *exposed to 150 cGy of X-ray radiation, more reduction (38.33) in the incidence of micronuclei was observed (p<0.001) (Figure 4).

**Frequency of micronuclei in human blood lymphocytes that were irradiated with 1.5 Gy X- radiation after administration of 600 mg GSE**

**Figure 5:**
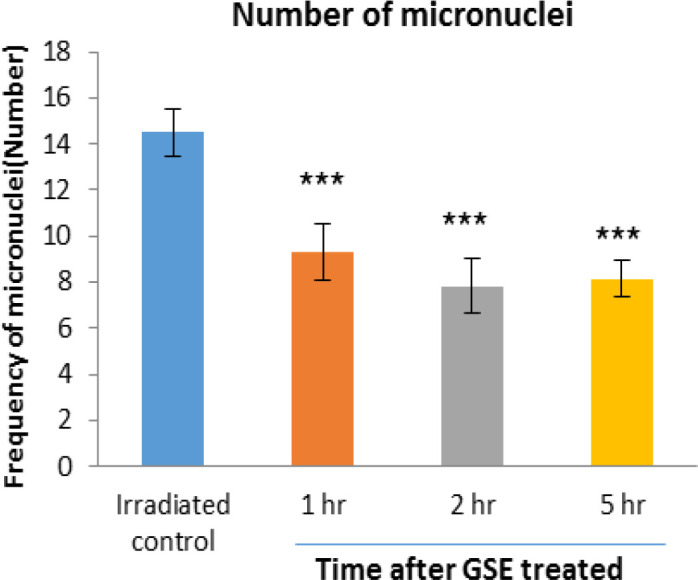
Mean number of micronucleated cells in human blood lymphocytes. Error bars indicate standard deviation(s) values. ***p<0.001 compared to irradiated control group. 1hr, 2hr and 5hr, indicate the groups, which were irradiation 1hr, 2hr and 5 hours after ingestion of 600 mg GSE

The frequency of micronuclei induced by 150 cGy of X-radiation in human lymphocyte in the control irradiated group was 14.5 and 1, 2 and 5 hr after 600 mg GSE ingestion were 9.33 (p<0.001), 7.83 (p<0.001), and 8.16 (p<0.001), respectively. The frequency of micronuclei found in GSE-treated cell groups was significantly lower than the control irradiated group. For the blood samples collected 2 hr after the oral GSE administration and *in vitro* exposed to 150 cGy of X-ray radiation, more reduction (46%) in the incidence of micronuclei was observed (p<0.001) (Figure 5).

**Frequency of micronuclei in human blood lymphocytes that irradiated with 1.5 Gy X- radiation after administration of 1000 mg GSE**

**Figure 6:**
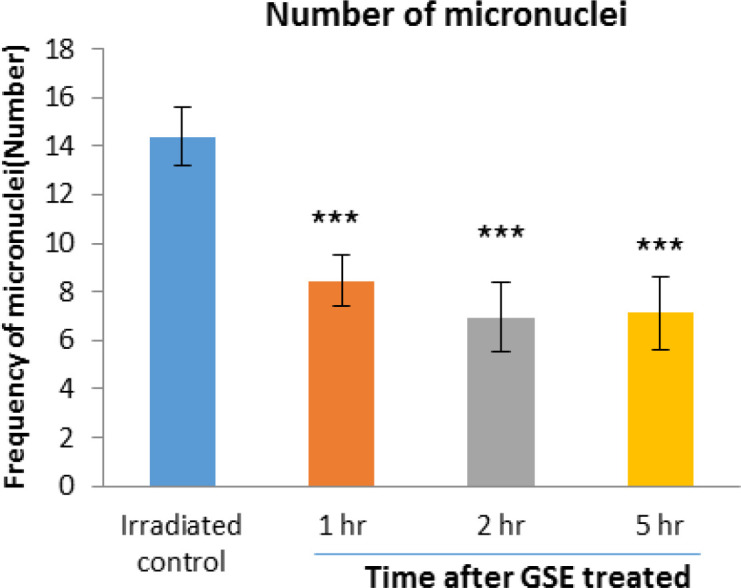
Mean number of micronucleated cells in human blood lymphocytes. Error bars indicate standard deviation(s) values. ***p<0.001 compared to irradiated control group. 1hr, 2hr and 5hr, indicate the groups, which were irradiation 1hr, 2hr and 5 hours after ingestion of 1000 mg GSE

The frequency of micronuclei induced by 150 cGy of X-radiation in human lymphocyte in the control irradiated group was 14.4 and 1, 2 and 5 hr after 1000 mg GSE ingestion were 8.45 (p<0.001), 6.95 (p<0.001), and 7.12 (p<0.001), respectively. The frequency of micronuclei found in GSE-treated cell groups was significantly lower than the control irradiated group. For the blood samples collected 2 hr after the oral GSE administration and *in vitro* exposed to 150 cGy of X-ray radiation, more reduction (51.73%) in the incidence of micronuclei was observed (p<0.001) (Figure 6). 

Comparison between the effects of various doses of GSE should be provided both in the results section and in the discussion section.

## Discussion

Micronucleus assay is a sensitive and effective method for assessing clastogenic effects of the chemicals and physical agents (Fenech et al., 2016b ▶). As it was obvious, irradiating blood sample with 1.5 Gy X-ray significantly increased the frequency of micronuclei, so ionizing radiation can induce DNA damages and chromosomal aberrations. Nearly 70 percent of radiation-induced chromosomal damages are produced by hydroxyl free radicals which are generated through indirect effect of ionizing radiations (Schmid, 1975 ▶). In addition, it was shown that a large number of single and double strand breaks is produced by these free radicals.

Because double strand breaks lead to chromosomal break and micronucleus formation (Fenech et al., 2016a ▶), it could be concluded that a large number of radiation-induced micronuclei are the result of free radicals generations. Bertucci et al. revealed that 2 Gy radiation increased micronuclei frequency in lymphocytes (Bertucci et al., 2016 ▶); Moreover, Hosseinimehr et al showed that irradiating humans lymphocytes with 2 Gy X-ray markedly increased the formations of micronuclei, which is in accordance with the present data (Hosseinimehr et al., 2009 ▶). A radioprotector agent can be used to protect humans from undesired exposure in nuclear accidents, medical imaging, radiotherapy, occupational settings, and space travels (Kamran et al., 2016 ▶; Smith et al., 2017 ▶). As mention amifostine is the only synthetic radioprotector that at efficient concentration has some side effects. Therefore, several studies investigated and explored different radioprotective agents with high efficiency and low toxicity. In this regard, natural compounds and plant extracts have attracted great attention and interest. 

It was revealed that antioxidant features of plant extract reduced the effects of ionizing radiation through their radical scavenging ability (Singha and Das, 2016 ▶). Grape seed extract (GSE), naturalizes intra cellular free radicals, and by donating hydrogen and sulfur improves DNA repair, thus reduces chromosomal damages (Nassiri‐Asl and Hosseinzadeh, 2016 ▶). GSE contains antioxidant agents including flavonoid, anthocyanins, proanthocyanidins and procyanidins, and poly phenolic compounds. The mentioned substances inhibit cell destruction by scavenging free radicals. It was shown that the potency of anthocyanins and poly phenolic compounds of GSE to protect DNA against radiation damages are 20 and 50 times higher than Vitamin C and E, respectively (Bagchi et al., 1997 ▶).

Several studies demonstrated radioprotective effects of some plant extracts including black tea, hespridin, *Polyalthia longifolia*, and *Malus baccata* (Ježovičová et al., 2016 ▶; Jothy et al., 2016 ▶; Wang et al., 2016 ▶; Karimi et al., 2017 ▶). These substances showed their radioprotective effects through their antioxidant and free-radical scavenging abilities. Another study showed that proanthocyanidin from grape seeds, has various beneficial properties including hepatoprotective effects as well as modulatory roles in age-related oxidative DNA damage (Balu et al., 2006 ▶). In addition, it was shown that hematopoietic system is one of the most sensitive tissue against low and acute doses of radiation (Hosseinimehr, 2007 ▶; Suryavanshi et al., 2015 ▶). Huang et al. showed that proanthocyanidin from GSE with its antioxidant activity, protects hematopoietic systems against radiation; authors showed that proanthocyanidin significantly reduced DNA strand breaks (DSBs) and apoptosis in human lymphocyte AHH-1 cells (Huang et al., 2016 ▶). Previously, by adopting micronucleus assay, the radioprotective activity of some plants in mouse bone marrow cells was studied (Targhi et al., 2016 ▶; Targhi et al., 2018 ▶; Shakeri-Boroujeni et al., 2016 ▶). It was shown that anthocyanin originated from grape increased the survival rate of irradiated mice and had a protective effect on hematopoietic tissues and the immune system (Hosseinimehr et al., 2007 ▶).

Cetin et al. (Cetin et al., 2008 ▶) showed that grape seed extract (as an antioxidant) treatment considerably increased the formation of antioxidants products in hepatocytes as shown by increased superoxide dismutase (SOD) and catalase activities and this effect may be due to the phenolic composition of grape seed extract and its antioxidant activity. Plants extract revealed radioprotective effects against cellular damage that caused by radiation. It was concluded that the radioprotective effect of herbal extract may be attributed to their radical-scavenging activity (Fischer et al., 2018 ▶). ROS generated by ionizing radiation, are scavenged by radioprotectors before they can interact with biochemical molecules, thus reducing the harmful effects of radiation. Radioprotective/antioxidative effects of various natural products were reported (Fischer et al., 2018 ▶). The antioxidant mechanisms of radioprotection and free-radical scavenging were attributed to flavonoids, orientin and vicenin (Li et al., 2011 ▶).

In the present study, it was revealed that administration of GSE at 100, 300, 600 and 1000 mg significantly decreased radiation-induced chromosomal damages (p<0.0001) in a dose-dependent manner without producing any side effects. These findings in human study are in good agreement with a previous animal study which found that GSE could reduce clastogenic and cytotoxic effects of gamma irradiation in mice bone marrow cells while all the mice could easily tolerate the extract (Targhi et al., 2018 ▶). 

Remarkable reduction in micronuclei frequency was due to the high antioxidant and free-radical scavenging abilities of GSE. The protective effect of GSE was dose- and time-dependent; the most protective effect was seen 2 hr following oral administrations of GSE 1000 mg (51.73% reduction in the incidence of micronuclei). 

The used doses of GSE are significantly lower than the toxic doses which is one of the advantages of using plants’ extract in comparison with chemical agents like amifostin. Therefore, it could be used at higher doses without causing toxicity. 

In the present study, the ingredients that caused the protective effects of the extract were not studied and it is needed to be investigated in future studies. 

The results revealed that oral administration of GSE before irradiation, leads to the reduction of DNA damages in human's blood lymphocytes; in addition, GSE did not show cytogenetic and toxic effects at different doses. Based on the results, it is suggested that oral administration of GSE before different medical interventions such as computed tomography, radiography, nuclear imaging, and radiotherapy, could be beneficial.
